# Prefrontal Cortex Lesions Impair Object-Spatial Integration

**DOI:** 10.1371/journal.pone.0034937

**Published:** 2012-04-26

**Authors:** Bradley Voytek, Maryam Soltani, Natasha Pickard, Mark M. Kishiyama, Robert T. Knight

**Affiliations:** 1 Helen Wills Neuroscience Institute, University of California, Berkeley, California, United States of America; 2 Department of Psychology, University of California, Berkeley, California, United States of America; 3 Department of Neurology, University of California San Francisco, San Francisco, California, United States of America; 4 School of Medicine, University of California San Diego, San Diego, California, United States of America; National Institute of Mental Health, United States of America

## Abstract

How and where object and spatial information are perceptually integrated in the brain is a central question in visual cognition. Single-unit physiology, scalp EEG, and fMRI research suggests that the prefrontal cortex (PFC) is a critical locus for object-spatial integration. To test the causal participation of the PFC in an object-spatial integration network, we studied ten patients with unilateral PFC damage performing a lateralized object-spatial integration task. Consistent with single-unit and neuroimaging studies, we found that PFC lesions result in a significant behavioral impairment in object-spatial integration. Furthermore, by manipulating inter-hemispheric transfer of object-spatial information, we found that masking of visual transfer impairs performance in the contralesional visual field in the PFC patients. Our results provide the first evidence that the PFC plays a key, causal role in an object-spatial integration network. Patient performance is also discussed within the context of compensation by the non-lesioned PFC.

## Introduction

The ability to navigate a complex visual world relies upon knowing both what an object is and where it is located. This capacity makes the difference between recognizing the red brake light on the motorcycle right in front of you from the red stoplight far ahead. Distinct ventral “what” and dorsal “where” pathways support object identification and location, nevertheless we are capable of seamlessly integrating object form with location information in a unified percept [1], [2]. Determining which brain regions are involved in integrating this information is a fundamental problem in visual cognition. A candidate area supporting this process is the prefrontal cortex (PFC), which shares reciprocal connections with both the ventral and dorsal processing streams [3] and maintains separate object and spatial domains [4]. Human electrophysiological and fMRI studies, as well as single-unit animal studies, support the notion that the lateral PFC is a conjunction area for visual information and location [5]–[7]. Interestingly, Simon-Thomas et al. [7], observed a performance boost during object-spatial integration compared to separate two-item object or two-item spatial tasks, suggesting that object and spatial information are processed in parallel, rather than in serial.

**Figure 1:**
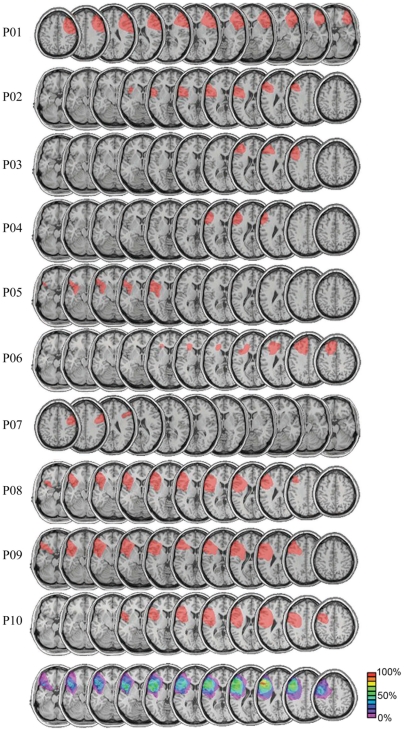
Patient MRIs. Lesion reconstructions are show for individual patients [n = 10], and we include a group average overlay (bottom). MRI reconstructions were obtained using MRIcro [32]. For the group average, patients with right hemisphere lesions [P01 and P07] were transcribed to the left hemisphere for display purposes. The color bar indicates the percent of patients with a lesion in a specific region. The area of greatest lesion overlap across the patients occurs in Brodmann areas 9 and 46, centered in the middle frontal gyrus.

To address whether the human PFC is a critical component in a distributed network supporting object-spatial integration, we tested ten patients with unilateral PFC lesions (see Figure 1 and Table 1) and age-matched controls who performed a speeded, lateralized visual object-spatial recognition task. By examining patients with unilateral brain lesions we were able extend the results of neuroimaging and single-unit research to address the causal role of the PFC in object-spatial integration with the hypothesis that, if the PFC is a critical component of the object-spatial integration network, patients with unilateral PFC lesions should show behavioral impairments relative to control subjects.

Furthermore, previous studies with human subjects [8]–[10] or monkeys [11] with unilateral PFC lesions have demonstrated a main effect of visual hemifield on performance wherein behavioral deficits are observed when stimuli are presented in the contralesional visual hemifield. Although patient behavior tends to be worse than controls, unilateral PFC lesions do not abolish higher cognition. Previous research from our lab suggests that intact, albeit impaired, cognitive functioning in patients with unilateral PFC lesions may be mediated in part by trial-by-trial compensation by the undamaged PFC [12]. In that experiment, Voytek et al. [12] expand upon previous work [8]–[11] by using scalp EEG to show that activity in the intact PFC increases with task demands only when the damaged hemisphere is challenged, and that this activity correlates with information transfer between the damaged and undamaged. While it was proposed that interhemispheric transfer of visual information from the damaged to the intact hemisphere was required for intact behavioral performance, that hypothesis could not be directly tested. Thus, in this experiment we include a secondary hypothesis wherein patients with PFC lesions would show exacerbated object-spatial integration deficits for contralesional stimuli when we manipulate the fidelity of information transfer between hemispheres. Based upon our previous findings related to functional recovery [12], we included in our design different timings of visual masking to explore the degree to which interhemispheric transfer of visual information from the damaged to the intact hemisphere mediates behavioral compensation.

**Table 1:** Patient Demographics.

	P01	P02	P03	P04	P05	P06	P07	P08	P09	P10
Age (yr)	76	71	63	64	60	45	53	43	47	64
Sex	F	M	M	F	M	M	F	M	F	F
Lesion etiology	stroke	stroke	stroke	stroke	stroke	stroke	HB	stroke	stroke	stroke
Lesion hemisphere	R	L	L	L	L	L	R	L	L	L
Lesion volume (cm^3^)	135.7	36.0	20.7	11.8	18.6	149.0	43.9	89.4	130.5	105.7
Time since lesion (yrs)	5	9	4	9	9	9	5	3	8	6
Lesioned regions (BA)	6, 8, 9	6, 9	6, 9	6, 9	6, 38	6, 8	6, 8	4, 6, 9	6, 8, 9	3, 4, 6, 9
	44, 45	44, 45	46	46	45, 47	9, 46	9, 46	43, 44, 45	10, 44, 45	39, 43, 44
	46, 47	46, 47			48			46, 47, 48	46, 47	45, 46, 48

Note: BA, Brodmann area; HB, hypertensive bleed.

## Results

**Figure 2:**
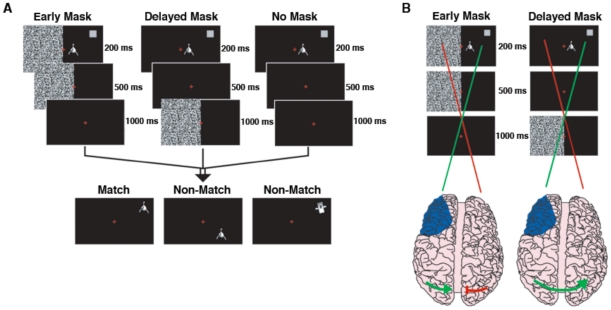
Behavioral Paradigm. (**A**) In all three conditions (early mask, delayed mask, and no mask) subjects were presented with an unidentifiable, non-verbalizable, black and white object and a gray location cue (see Materials and Methods for details). (**B**) Schematic of the main hypothesis. In the early mask condition, the mask adds noise during the processing of the visual object and spatial cue by the non-lesioned hemisphere, reducing the fidelity of the transcallosal transfer of visual information (disconnected green/red line over visual cortex). In the delayed mask condition, however, task-relevant visual information crosses the corpus callosum before the mask appears, allowing the non-lesioned hemisphere to assist in object-spatial recognition (intact green line over visual cortex). Blue shading illustrates the location of the subjects’ lesions.

To examine the role the PFC plays in object-spatial integration we tested a group of patients with unilateral PFC lesions (Figure 1) and controls on a lateralized visual object-spatial integration task (Figure 2). Briefly, the task involved a short presentation (200 ms) of a non-verbalizable object (a “Greeble”) [13] and a location marker (grey square). After a 1500 ms delay period a second object was presented in a different location. Only if the second object was identical to the first and was located in the position indicated by the original grey square would the trail be considered a “match”. Some trials included a 500 ms white noise mask during the delay period (see Methods for full details).

### Effect of lesions on object-spatial integration

**Figure 3:**
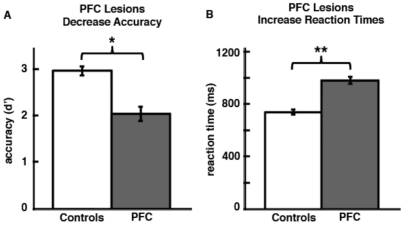
Object-Spatial Behavioral Results. (**A**) Patients showed an overall impairment in object-spatial integration resulting in decreased stimulus sensitivity (d’) across all trials and conditions. (**B**) Similarly, patients showed an overall response impairment resulting in increased reaction times across all trials and conditions. Error bars indicate SEM. (*), significant difference with p = 0.0032; (**), significant difference with p<0.0005.

As predicted in our main hypothesis, there was a main effect of group on accuracy wherein the PFC patients performed worse than controls [F(1,18)  = 13.07, p = 0.002] (Figure 3A), however there was no hemifield by group interaction [F(1,18)  = 1.18, p = 0.29]. There was also a main effect of age [F(1,18)  = 5.82, p = 0.027] and response type [F(1,18)  = 7.96, p = 0.011] such that older subjects performed worse and both controls and PFC subjects were more impaired for non-match trials.

Similar to the accuracy results, overall reaction times were slower in the PFC group [F(1,18)  = 20.06, p<0.0005] (Figure 3B) and there was a main effect of age [F(1,18)  = 10.94, p = 0.004], with older subjects responding slower as well as a trend toward a main effect of response type between location non-match and object non-match trials on RT (F_1,19_ = 3.90, *p* = 0.063). However, unlike the accuracy results, there was also a trend toward a group by hemifield interaction [F(1,18)  = 3.75, p = 0.069] with PFC patients responding slower for contralesional stimuli and control subjects performing equally for both hemifields. Of note, the PFC group performed well above chance levels (*post hoc* one sample t-tests for each hemifield [p<0.005 for both comparisons]). This suggests that, although unilateral PFC lesions impair behavior, unilateral lesions are not sufficient to abolish object-spatial integration.

Within the control group, there was only a main effect of age on accuracy [F(1,9)  = 6.75, p = 0.029] and on reaction times [F(1,9)  = 30.79, p<0.0005]. Within the PFC group, there were no main effects or interactions on accuracy, however there was a three-way interaction between hemifield, mask, and response type for reaction times [F(2,14)  = 4.75, p = 0.027]. To test the hypothesis that the intact hemisphere compensates for unilateral PFC damage, we included in our design a visual mask presented to the hemifield opposite the task stimulus during two time windows: a mask was presented either early, in conjunction with the task stimulus (0–500 ms after stimulus onset), or later during the delay period (500–1000 ms). There was also a no-mask condition where the object-spatial stimulus was presented without any concurrent masking of the opposite hemifield (Figure 2B). The masks were used to manipulate the fidelity of information transfer between the hemispheres, with the hypothesis that the early mask specifically would reduce the fidelity of the relevant information that crosses into the opposite hemisphere. In contrast, the delayed mask would serve to control for the effects of the distractibility of the mask while allowing for the visual information to transfer between hemispheres more completely.

Findings from an earlier experiment [12] suggest that interhemispheric transfer of task-relevant information might be a critical component of compensatory support by the intact hemisphere. We incorporated a visual mask into our design to specifically test the hypothesis that the intact PFC compensates for the damaged hemisphere when the information presented to the lesioned hemisphere transfers to the non-lesioned hemisphere via the corpus callosum [14], [15] whereby the non-lesioned hemisphere assumes task control and assists in stimulus processing [12].

### Examining the role of compensation in patient performance

**Figure 4:**
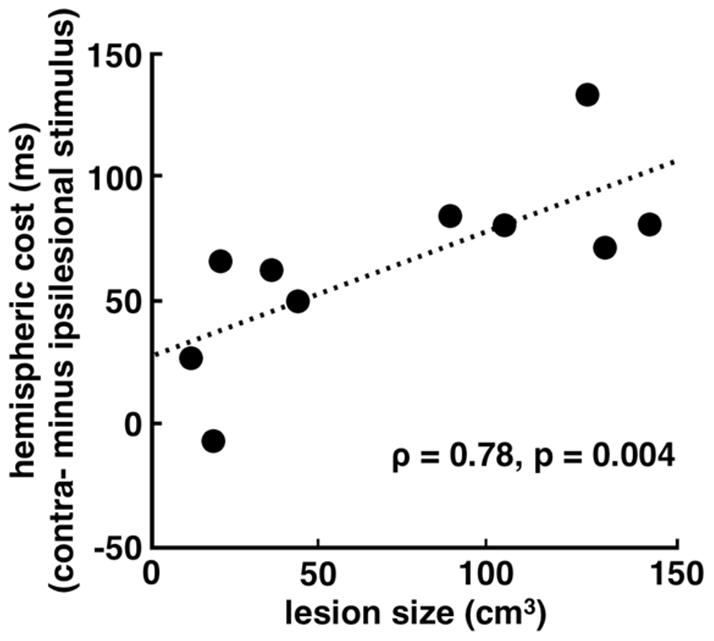
Lesion Size Correlates with Patient Behavior. Patients with larger lesions show a greater behavioral difference between ipsilesion and contralesion stimuli during the mask condition. When a mask is present, patients with the largest lesions show the most slowing when responding to contralesional stimuli (Spearman’s ρ = 0.78, p = 0.004).

To examine the nature of the three-way interaction in the PFC patients on reaction time, and the hypothesis that a visual mask presented to the intact hemisphere concurrently with the task-relevant stimulus would affect compensatory functions by the intact hemisphere, we performed a series of *post hoc t*-tests to examine the effect of hemisphere on behavioral performance. We observed that the PFC patients had slower reaction times for contralesional stimuli only when processing by the intact hemisphere was disrupted using an early mask [t(9) = 5.50, p<0.0005]. Interestingly, patients with larger lesions showed a greater difference between contralesional and ipsilesional stimulus reaction times when there was an early mask present (Figure 4; Spearman’s ρ = 0.78, p = 0.004). That is, patients with larger lesions took longer to respond to contralesional – compared to ipsilesional – stimuli, which suggests that the degree of compensation required for correct behavioral performance is linked to the amount of PFC damage.

**Figure 5:**
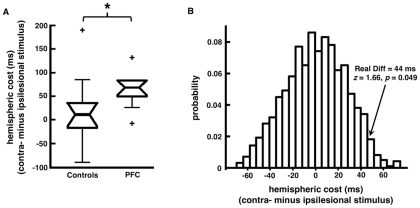
Effects of Early Mask on Patient Performance. (**A**) Box plot comparing control (left) and patient (right) hemispheric cost during the mask condition (contralesional minus ipsilesional). Nine out of the ten participants show this hemispheric cost whereas control subjects show no real bias. (**B**) We confirmed group differences by way of resampling statistics (see **Methods**), which confirm that the hemispheric behavioral asymmetry is greater in patients compared to controls (z = 1.66, p = 0.049). (*), significant difference with p = 0.050.

We found that 9 of our 10 subjects showed increased reactions times in response to contralesional stimulus presentation when a concurrent (early) visual mask was delivered at the time when information would normally be transferred between the visual hemispheres (see Figure 5A and Table 2). This disruption manifested as an average of a 65 ms response time deficit within the PFC group for masked stimuli presented to the contralesional hemifield compared to stimuli presented to the ipsilesional hemifield. This effect was not seen in control subjects (p = 0.050, one-tailed t-test; p = 0.049, resampling statistics, Figure 5A and B) nor was it seen in any other condition within the PFC group (p>0.10 Bonferroni-adjusted for all comparisons).

**Table 2:** Summary of Reaction Times.

	Control: mean (SEM)		PFC: mean (SEM)	
	Left	Right	Ipsilesional	Contralesional
No Mask				
Match	714 (45)	712 (37)	929 (47)	958 (42)
Non-match	774 (46)	762 (44)	1038 (65)	1055 (63)
Delay Mask				
Match	726 (42)	731 (38)	910 (45)	946 (56)
Non-match	748 (42)	752 (41)	1008 (69)	1046 (65)
Early Mask				
Match	713 (40)	733 (39)	904 (43)	969 (39)*
Non-match	755 (40)	764 (44)	1028 (76)	1047 (64)

Note: SEM, standard error of the mean; Hemispheric differences: *P<0.0005.

Furthermore, reaction times were slower in both groups for non-match compared to match trials. This suggests that subjects required more time to evaluate object-spatial information when the object was not located in cued spatial region. One plausible explanation for this effect would be that, during object-spatial disjunctions, subjects had to perform and an extra processing step. Such a step would fit with the notions of conflict monitoring, a process that depends on the anterior cingulate cortex [16] and engages different neural systems when relevant endogenous information conflicts with an exogenous test stimulus [17]. Patients with unilateral PFC lesions show intact conflict monitoring, albeit with slowed response times [18], a pattern that fits our current findings given that response condition did not interact with hemisphere of presentation or mask type. The conflict monitoring processing delay induced by non-match trials may have engaged more neural processing systems and may have given the PFC patients a performance boost, possibly due to reengaging compensatory processes during the extra delay. If this hypothesis were true, PFC subjects who showed a greater discrepancy between non-match and match trial reaction times would show less of a contralesional stimulus cost during mask trials. In other words, the PFC patients that take longer to respond would show less of a hemispheric effect, possibly due to a greater engagement of compensatory processes. As hypothesized, we found that PFC patients who had slower reaction times for non-match compared to match trials showed less of an accuracy difference between contralesional and ipsilesional stimuli during those same trials [Spearman’s ρ = 0.65, p = 0.042].

## Discussion

We investigated the role of the intact PFC in supporting object-spatial integration in patients with unilateral PFC damage. During the integration phase, initially separate streams of object and spatial information are fluidly combined into a single unified percept within a few hundred milliseconds. Primate studies have shown that the PFC exhibits differential neural firing patterns to “what”, “where”, and “what-where” combined information [4], [6]. While the classic distinction between a dorsal “what” and ventral “where” pathway has been shown to be an overly simplified model [19]–[22], human electrophysiology and fMRI studies have demonstrated that the PFC might act as a conjunction area for object-spatial integration at the “top” of this visual processing hierarchy [5], [7]. We show that humans with unilateral PFC lesions have impaired ability to integrate object and spatial information. Despite these impairments, subjects still performed above chance levels. Although we cannot entirely rule out the possibility that patients may have saccaded toward the stimuli of interest due a lack of quantitative eye movement recording, the short duration of stimulus presentation (200 ms) – combined with the significant group behavioral differences – suggests that visual monitoring of eye movements was mostly successful. If patients were saccading to the stimuli of interest, we would expect this reduction of selective stimulation of the damaged hemisphere to result in an overall improvement of their behavioral performance relative to controls, which was not observed.

Nevertheless, it would seem that – although PFC lesions impair object-spatial integration – a unilateral lesion is not sufficient to abolish this cognitive ability. It has been shown that neurons within the PFC primarily represent the contralateral visual field [19], [20], yet surprisingly, despite the lateralization of the visual stimuli, there was no effect of hemifield of presentation on patient behavioral performance in our experiment. This is in contrast to previous reports from our lab and others that demonstrate lateralized, contralesional working memory [8], [11], [12] and attention [9], [10] deficits in patients with unilateral PFC lesions. While this lack of a hemispheric effect in the PFC group (for non-match trials and no-mask or delay-mask match trials) may be due to patients saccading to the stimulus of interest, the finding that patients with longer reaction times were more accurate for contralesional stimuli suggests that there may be a relationship between response time and compensation that merits future research.

We posited that the intact, non-lesioned PFC assisted the damaged hemisphere to support object-spatial integration. We tested this possibility by examining the effect of a concurrently presented visual mask on behavioral outcomes. We found evidence that the intact PFC plays a crucial role in cognitive compensation. We showed that PFC patients were slower to respond to contralesional stimuli only when we interfered with the transfer of visual information between the two visual cortices with a visual mask presented to the intact hemisphere. In the delayed mask condition, the mask did not appear until 500 ms after the onset of the object-spatial stimuli, long after the information would have transferred from the damaged to intact hemisphere.

It is important to reiterate that we observed an overall behavioral deficit in object-spatial integration in patients with unilateral PFC lesions. These deficits manifested both as an overall decrement in accuracy as well as increased reaction times. These data provide, for the first time, causal neuropsychological evidence that the lateral PFC is a key node in the network employed for the integration of object form and spatial location. We must note, however, that patients with PFC lesions in general have attention and working memory deficits, so although we show that object-spatial integration is impaired in these subjects, we also cannot rule out the possibility that a generalized executive dysfunction underlies this observation. Although we have specifically chosen to examine object-spatial integration to extend our previous work that shows these types of information are processed in parallel [7], the metaphor of a dorsal “what” and ventral “where” pathway is oversimplified [21]. For example, it has been shown that the lateral occipital complex in the ventral visual pathway encodes both object and location information [22] but that, though object category and location information are jointly encoded in many visual regions [23], [24], object and spatial information may potentially still be considered functionally separate [24].

Our secondary results support the notion that callosal transfer to the non-lesioned hemisphere contributes to the patients’ abilities allowing them to conduct goal-directed behaviors successfully, even after suffering unilateral brain damage [12]. Research on macaques suggests that the posterior corpus callosum is necessary for interhemispheric transfer of visual information, but once the information is transferred, long-term retrieval is mediated by PFC communication and is not affected by a posterior corpus callosum split [25]. Electrophysiological evidence shows that information transfers transcallosally between hemispheres within 15–20ms of lateralized stimulus presentation [15]. Here we show how a lateralized visual paradigm can be used to assess the role of the PFC in object-spatial integration. Furthermore, we show that masking the intact visual cortex impairs performance in patients with unilateral PFC lesions, supporting the contention that intact homologous brain regions support cognitive functioning after brain damage. We cannot specifically address the nature of this compensation; functional recovery may arise from post-lesion neural reorganization such as axonal sprouting [26] and/or neurogenesis [27]. And while we cannot rule out that other forms of compensation such as processing by perilesion brain regions [28], unmasking [29], or diaschisis reversal [30] may have supported patient behaviors, we designed our experiment to specifically mask compensatory processing by the intact PFC via interhemispheric information transfer. So while other factors such as incomplete injury to a hypothetical “critical region” of object-spatial integration in the PFC may account for partially intact patient performance, enhanced patient deficits during the early mask period supports the hypothesis that behavioral compensation is partly due to interhemispheric transfer of visual information to the intact hemisphere.

## Methods

### Participants

Ten patients with unilateral damage to the lateral PFC (8 left and 2 right hemisphere lesions, aged 43–76; see Figure 1 and Table 1 for details) and eleven age-matched controls (aged 43–76) were tested. All subjects provided written, informed consent to participate in the study and were recompensed. All patients were in the chronic stage, at least one year post stroke at the time of testing. The research was approved by the UC Berkeley Committee for the Protection of Human Subjects. Subjects were tested individually on a desktop computer in a dark, soundproof booth. They sat ∼90 cm from the computer monitor.

### Behavioral task

During the tasks, subjects fixated on a red cross in the center of a computer screen. An experimenter visually monitored eye movements to ensure that subjects maintained fixation and minimized saccades during the task. Subjects’ eye position was monitored on every trial and trials where subjects made saccades were excluded from analyses. An unidentifiable object (a “Greeble”) [7], [13] was presented 3 degrees from the fixation cross and paired with a location cue which appeared in one of seven different locations on the screen in the same hemifield (200 ms duration to minimize saccadic eye movement, i.e., foveating to the stimuli). Stimuli consisted of a gray location cue (∼4.0×4.0 cm), five different, unidentifiable, non-verbalizable, black and white objects (∼5.0×5.0 cm), and a static white noise visual mask flashing at a rate of 16 Hz. After a 1000 ms delay, subjects decided whether the test object was the same as the initial object and if it appeared in the same spatial location as the initial cue (integration effect) by pressing one of two buttons on a computer keyboard. During the test phase, if the test object was the same as the original object and appeared at the initial location of the cue, the trial was a match. If either the object or the location was different, the trial was a non-match. If no response was made after 2000 ms, the next trial would begin. Trials were randomized to either the left or the right visual field with equiprobability. There were three mask conditions: early mask, delayed mask, and no mask. In the early mask condition, the noise mask was presented for 500 ms in the field opposite to the concurrently delivered object-spatial stimulus to reduce the fidelity of interhemispheric transfer of the visual stimuli to the intact hemisphere. In the delayed mask condition, the mask was presented for 500 ms following a 500 ms delay after the stimulus onset. This condition served as a control for the potentially distracting effects of the mask. In the no mask condition, patients were presented with only the object and location cues. E-prime (Psychology Software Tools, Inc., Pittsburgh, PA) was used for stimulus presentation and data analysis was performed using SPSS® (Rel. 16, Chicago: SPSS Inc.).

### Data analysis

Accuracy was quantified using a d statistic, which considers both correct responses and false alarms, to account for response bias [31]. For d’ analyses, “hits” are correct responses and “false alarms” are incorrect responses not taking into account “misses” (trials that fall outside the 2000 ms response time window). Thus, for trials in which the correct answer was match, then a non-match response would be a false alarm, and vice versa. Statistical comparisons were run using multiple repeated measures ANCOVA on reaction time and accuracy separately with mask condition (no mask, early mask, or delayed mask), hemifield of presentation (ipsilesional/left or contralesional/right), and response type (match or non-match) as the within-subjects factors, and group (control vs. PFC) as the between-subjects factor. We included age and (for the patient-only analyses) lesion size as the covariates in our ANCOVA. The variables were included as covariates because they both covary with the behavioral measures of interest such that reaction times increase with age and, for patients, compensatory processes are affected by lesion size (see Figure 4). Thus we wanted to rule out the possibility that differences between subjects across these factors were masking the effects of interest. Each subject had a least 50 correct trials per condition, hemifield of presentation, and response type combination.

For the resampling analysis shown in Figure 5B, we calculated a hemispheric cost index (contralesional minus ipsilesional reaction times) for each subject. In order to confirm the one-tailed t-test analysis, we performed a resampling analysis wherein we combined this hemispheric index for both groups, and then drew 2 groups of random data from this joint pool: 10 surrogate patients and 11 surrogate controls. We then took the difference of the means between these two surrogate groups and repeated this process 1000 times to get the empirical distribution of possible group differences from the data. Because of the central limit theorem, the distribution of these surrogate differences will approach normal. We can then compare the real difference against this empirical difference by calculated the z-score and its associated p-value.
